# Ability of Vital and Fluorescent Staining in the Differentiation of *Schistosoma haematobium* Live and Dead Eggs

**DOI:** 10.3390/medsci7040064

**Published:** 2019-04-23

**Authors:** Peter O. Forson, Patience B. Tetteh-Quarcoo, John Ahenkorah, Robert Aryee, Esther N. Okine, Emmanuel Afutu, Georgina I. Djameh, Jeffrey Agyapong, Abraham K. Anang, Patrick F. Ayeh-Kumi

**Affiliations:** 1 Department of Medical Microbiology, School of Biomedical and Allied Health Sciences, College of Health Sciences, University of Ghana, Accra 00233, Ghana; petsonbiomed2015@yahoo.com (P.O.F.); bobby200055@gmail.com (R.A.); eafutu@ug.edu.gh (E.A.); pfayeh-kumi@ug.edu.gh (P.F.A.-K.); 2 Department of Anatomy, School of Biomedical and Allied Health Sciences, College of Health Sciences, University of Ghana, Accra 00233, Ghana; jahenkorah@ug.edu.gh; 3 Department of Physiology, School of Biomedical and Allied Health Sciences, College of Health Sciences, University of Ghana, Accra 00233, Ghana; 4 Central Laboratory Services, Korle-Bu Teaching Hospital, Korle-Bu, Accra 00233, Ghana; gyister7@yahoo.co.uk; 5 Department of Parasitology, Noguchi Memorial Institute for Medical Research, College of Health Sciences, University of Ghana, Accra 00233, Ghana; ginabel200@gmail.com (G.I.D.); jephrey14@gmail.com (J.A.); KBosompem@noguchi.ug.edu.gh (A.K.A.)

**Keywords:** Hoechst 33258, schistosomiasis, *Schistosoma haematobium*, staining, viability, vital stains

## Abstract

This study reports (for the first time) the staining ability of vital (0.4% trypan blue and 1% neutral red) and fluorescent (Hoechst 33258) dyes to differentiate between live and dead *Schistosoma haematobium* (*S. haematobium*) eggs in human urine samples. Since *S. haematobium* egg is important in disease pathology, diagnosis, transmission, and drug development research, it is essential to be able to easily distinguish live eggs from dead ones. Staining is considered a way of enhancing the identification of live and dead eggs. Urine samples from school children were examined for the presence of *S. haematobium* eggs. Vital and fluorescent dyes were used to stain the samples that contained *S. haematobium* eggs, after which they were observed using light and fluorescent microscopes, respectively. The Hoechst 33258 provided a good staining outcome for differentiation between live and dead eggs, followed by 0.4% Trypan blue. Regarding the 1% neutral red stain, even though it provided some evidence of which egg was alive or dead, the distinction was not very clear; therefore, it could be useful when used in combination with other stains for egg viability determination. The benefits of this study will include assessing the effect of drugs on *S. haematobium* eggs in Schistosomiasis research.

## 1. Introduction

Globally, schistosomiasis remains an important disease of public health concern, with over 200 million cases reported each year [1]. However, about 85% of the reported annual cases occur in sub-Saharan Africa, and over 150,000 deaths are attributable to chronic infection with *Schistosoma haematobium* (*S. haematobium*) in this region [2,3]. *Schistosoma haematobium* is the main species involved in urinary schistosomiasis, where eggs are laid by the adult worm living in the venous plexus of the bladder [4]. The eggs are either shed into the environment through urine or retained in host tissues where they provoke granulomatous inflammation, ulceration, and pseudo-polyposis of the vesical and ureteral walls [5,6]. Haematuria is a very common sign of infection, but proteinuria dysuria, and pollakisuria are among other notable signs [5]. Kidney failure due to urinary tract scarring and deformity of the ureters and bladder have been associated with *S. haematobium* infection [5,6]. *Schistosoma haematobium* has also been implicated in squamous-cell carcinoma of the bladder [7]. 

Research involving schistosome eggs would contribute immensely in various ways towards schistosomiasis research, since it is obvious that the eggs are a major cause of morbidity in schistosome infections [8]. One example of such research would be being able to clearly differentiate between live (viable) and dead (non-viable) eggs. Knowing the viability of the eggs would be important for numerous reasons, including transmission/control, schistosomiasis drug development research, disease pathology, diagnosis, and prognosis. First, being able to clearly identify if a sample were made up of live or dead *S. haematobium* eggs would help in ascertaining if that patient could contribute to the transmission of urogenital schistosomiasis, and hence control strategies that would block transmission and reduce the disease burden that would be put in place. Second, during in vitro studies, if one were able to easily differentiate between live and dead eggs, it would contribute significantly to research on drug development that targets the eggs. It could also be helpful for ascertaining whether a schistosomiasis drug that was not originally designed to target the eggs might be having an effect on the viability of the eggs. Third, since the eggs are the main cause of pathology of the disease [8], knowing whether there are more viable eggs in a patient would help establish the extent of pathology, eventually helping with diagnosis and prognosis. Therefore, having staining methods that are simple and economical for a neglected tropical disease like schistosomiasis, which effectively helps to differentiate between live and dead eggs, makes sense, considering that schistosomiasis is a major problem in less privileged locations [2,3].

The standard for diagnosis of *S. haematobium*-schistosomiasis is the microscopic detection of oval-shaped parasite eggs with a terminal spine present in urine [9]. The eggs can be classified as live, dead, shells, and granulomas, based on morphological characteristics, and are enhanced by staining [10]. This differentiation of live and dead eggs by staining has been beautifully demonstrated by Sarvel et al. [10], using eggs of *Schistosoma mansoni* (*S. mansoni*) obtained from the intestines of infected mice. Due to the importance of *S. haematobium* eggs in disease pathology, diagnoses, transmission, and drug development research, it is important to be able to distinguish live eggs from dead ones. This study therefore reports (for the first time) the staining ability of vital (0.4% trypan blue and 1% neutral red) and fluorescent (Hoechst 33258) stains to differentiate between live and dead *S. haematobium* eggs in human urine samples collected from infected children.

## 2. Materials and Methods

### 2.1. Study Site and Urine Sample Collection

Clean, dry, leak-proof, wide-mouthed containers were given to school children living in the Zenu and Weija communities to provide urine samples, after obtaining consent from the children and their parents, guardians, and teachers.

The Zenu community is situated in the southern part of Ashiaman municipality, which is a suburb of Accra, the capital city of Ghana. Its geo-graphical coordinates are 5°42′0″ North, 0°20′0″ West [11]. The presence of a lake in the community might have attracted most of the settlers. Weija is a community that lies in the South Western part of Accra. It contains are two main rivers, namely, the Ponpon and Densu rivers, which drain the Weija community. Densu is one of the main sources of water supply to more than half of the population of the Accra Metropolis. These two locations, namely, Zenu and Weija, have been considered as endemic for urogenital schistosomiasis [11,12] and thus were selected for the current study in order to increase the chances of obtaining *S. haematobium*-positive samples.

Using a clearly labelled 50 mL container (Sree Sai Industries, Bengaluru, India), urine samples were collected from each enrolled child between 10:00 am and 12:00 pm for maximum yield [13]. The urine samples were then transported on ice, within 45 to 60 min, to the Parasitology Laboratory of the Medical Microbiology Department, University of Ghana, for laboratory investigation [13]. 

### 2.2. Preparation of Urine Samples

For every ten milliliters (10 mL) of the collected urine samples that was poured into centrifuge tubes and spun at 3000 revolutions per minute (rpm for 3–5 min, the supernatant was discarded until the one milliliter (1 mL) mark. Fifty microlitres (50 µL) of the sediment was transferred onto clean glass slide. This was done to detect the presence of *S. haematobium* ova, which is described by its characteristic oval shape and a terminal spine. Samples that had *S. haematobium* ova were subsequently selected for the staining (vital staining and fluorescent staining) assessment. 

### 2.3. Vital Staining (Trypan Blue and Neutral Red)

The vital stains (0.4% trypan blue and 1% neutral red) were used separately. Ten microliters (10 µL) of the 0.4% Trypan blue was added to every 500 µL positive *S. haematobium* urine suspension [10] and four slides (of 50 µL each) produced from it (to increase the probability of obtaining live and dead eggs), after which the slides were incubated in petri dishes containing moist cotton for 5 min.

Also, similar to the trypan blue preparation, 10 µL of 1% neutral red was added to every 500 µL of positive *S. haematobium* urine suspension [10], after which four slides (of 50 µL each) were produced from it and incubated in petri dishes containing moist cotton, for 5 min. After 5 min, it was observed under the optical light microscope (Leica Galen III, catalogue e no. 317506, serial no. ZG6JA4, Cambridge, UK) using the ×10 and ×40 objective to check for stain retention in the eggs for classification as either dead or alive. 

Interpretation of results was based on information from literature that trypan Blue dye is able to stain only cells considered to be dead, while neutral Red dye is able to stain living cells [10,14]. Thus, in this study, it was expected that dead *S. haematobium* eggs would homogenously retain the 0.4% trypan blue dye, producing a blue colouration, while the live ones would not. In contrast, live *S. haematobium* eggs (viable) showed total stain retention (red colouration) of the 1% Neutral red, while the dead ones did not. 

### 2.4. Fluorescent Staining (Hoechst 33258)

Fluorescent Hoechst 33258 staining was employed in the current study as previously described by Sarvel et al. [10]. The fluorescent probe Hoechst 33258 was diluted in 0.85% saline, and 1 mg/mL of the stock solution was obtained. Ten microliters of the probe was added to every 1 mL of egg suspension, and slides made from them were incubated at room temperature for 20 min prior to examination. Using a reading filter of 460 nm, the examination was done under the fluorescent microscope (Olympus, BX-530, Tokyo, Japan). According to Sarvel et al. [10], when labelled with the probe Hoechst 33258, fluorescence is a staining characteristic of dead eggs, and this was expected in the current study.

### 2.5. Quality Control

In order to ensure that accurate and credible results were obtained, positive and negative controls were performed for the experiment. The positive control consisted of a known human urine sample that contained mature eggs with characteristic movement of their miracidium, the flame cells, as well as the hatching of eggs. For the negative control, the urine sample containing the eggs was frozen at −20 °C for one day, after which the suspension with eggs was thawed. The dyes were added to suspensions of both the positive and negative control samples, alongside the test samples. 

### 2.6. Ethical Approval and Consent to Participate

This work was conducted in accordance with the Declaration of Helsinki (1975). Before undertaking the research, approval of the Ethical and Protocol Review Committee of the College of Health Sciences, University of Ghana was obtained, with identification/reference number CHS-Et/M.3 P 3.5/2016-2017. Urine samples were collected from study participants with consent from children and their parents, guardians, and teachers.

## 3. Results

### 3.1. Viability by Vital Staining

**Figure 1:**
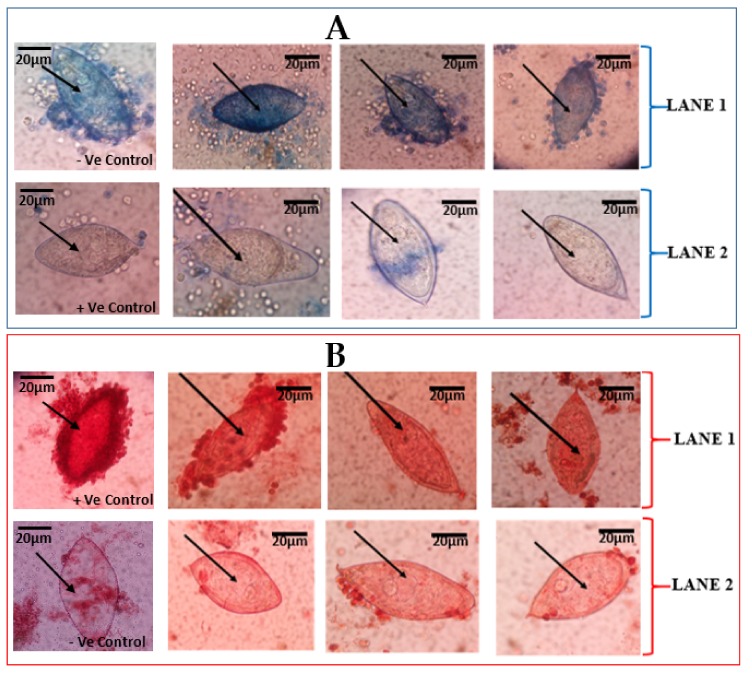
Vital staining of *Schistosoma haematobium* eggs from urine of school children. (**A**) *S. haematobium* eggs stained by 0.4% trypan blue observed at ×40 Objective lens: (**A**) LANE 1 shows eggs that retained the 0.4% trypan blue, indicative of dead *S. haematobium* eggs. LANE 2 shows eggs that did not retain of 0.4% trypan blue stain, indicative of live eggs. The first slides of LANES 1 and 2 show –Ve (negative) and +Ve (positive) controls, respectively. (**B**) *S. haematobium* eggs stained with 1% neutral red and observed at ×40 Objective lens. LANE 1 shows eggs with retention of 1% neutral red, indicative of live eggs of *S. haematobium*. LANE 2 shows eggs with non-retention of 1% neutral red stain, indicative of dead eggs. The first slides of LANES 1 and 2 show +Ve (positive) and -Ve (negative) controls, respectively.

The eggs that were effectively stained by the trypan blue were considered dead, whiles those that were not stained were considered to be morphologically alive (Figure 1A). Incubation time played a critical role in distinguishing these differences between the eggs. The neutral red stain was able to stain (red colouration) eggs considered to be viable. Meanwhile, the eggs considered to be dead (that were expected not to stain) stained partially, showing light red colouration (Figure 1B).

### 3.2. Viability by Fluorescent Microscopy

**Figure 2:**
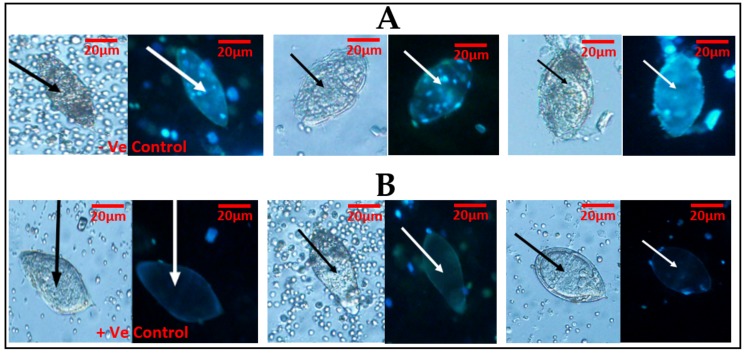
*Schistosoma haematobium* ova was observed under ×20 objective lens using light microscopy first, followed by fluorescent microscopy, with the cell stain (Hoechst 33258): (**A**) two eggs in light microscopy followed by fluorescent microscopy using Hoechst 33258 and showing fluorescence (blue), indicative of dead eggs. (**B**) A lane demonstrating two eggs in light microscopy, followed by fluorescent microscopy using Hoechst 33258 and demonstrating no fluorescence, indicative of live eggs. The first slides of (**A**) and (**B**) show –Ve (negative) and +Ve (positive) controls, respectively.

The fluorescent probe (Hoechst 33258) used to stain the eggs was able to differentiate live eggs (viable) from dead eggs (non-viable). The dead eggs were able to show fluorescence in blue (Figure 2A), while the live eggs did not show fluorescence (Figure 2B). Meanwhile, in one of the samples, there was an observation of an egg that seemed to be viable but showed fluorescence. 

## 4. Discussion

This study reports (for the first time) the staining ability of vital (0.4% trypan blue and 1% neutral red) and fluorescent (Hoechst 33258) stains to differentiate between live and dead *S. haematobium* eggs isolated from human urine samples and collected from infected children.

Various vital dyes, including trypan blue, methylene blue, resorufin, erythrosin B, nigrosine, eosin, safranin, propidium iodide and 7-aminoactinomycin D, have been introduced to identify cell viability [14,15,16,17,18]. Among these, trypan blue is widely used for viable cell counting with bright-field optics [16]. The fluorescent label Hoechst 33258 (bisbenzamide) (2,4-hydroxyphenyl-5,4-methyl,1-piperazine-2,5-bis*H*-benzymidazol) is a hydrophilic and fluorescent probe that is permeable to the nuclei and binds to the minor groove of double stranded DNA of cells. It has been reported to be a cell stain that can be used for the visualization of viable cells [19], and has often been employed as a substitute for other dyes such as 4′,6-diamidino-2-phenylindole (DAPI) due to its low toxicity and the enhanced viability of its cells. Fluorescent Hoechst 33258 has been considered a useful tool for differentiation between live and dead eggs by Sarvel et al. [10]. 

In this study, vital stains (0.4% trypan blue and 1% neutral red) as well as fluorescent label Hoechst 33258 were used to distinguish dead eggs of *S. haematobium* from live eggs, similar to what was done in *S. mansoni* by Sarvel et al. [10]. 

The vital stains, 0.4% trypan blue and 1% neutral red, were able to distinguish live eggs from dead ones, to some degree. Dead eggs were clearly stained (blue colouration) by the 0.4% trypan blue, while live eggs did not retain the trypan blue stain. Regarding the 1% neutral red, live eggs were clearly stained (red colouration), while dead eggs were partially stained, thus allowing differentiation of live eggs from dead eggs only to a certain degree. The fluorescent dye (Hoechst 33258) presents a blue fluorescence in dead cells, since the Hoechst 33258 solution has been described as a reagent for the fluorescent staining of DNA and nuclei, including dead cells [10,14].

It was observed in the current study that, among the vital stains, even though the 1% neutral red stain provided some evidence of which egg could be viable or dead, the distinction was not very clear, thus partially agreeing with the assertion by Sarvel et al. [10] that vital dyes might not be efficient in differentiating between live and dead eggs. It would therefore be more useful for 1% neutral red to use in combination with other stains (like the 0.4% trypan blue and fluorescent Hoechst 33258) for egg viability determination. Our findings also support the conclusion by Sarvel et al. [10] that the fluorescence Hoechst 33258 can be considered a good tool for differentiation between live and dead schistosoma eggs, since it showed the best staining ability in the current study. 

Even though it is expected that dead eggs would fluoresce [10], in the current study, the existence of the single seemingly live egg, which showed fluorescence, could be due to the stage of the egg [10]. This is because, even though the current study did not investigate the performance of the stains on different stages of *S. haematobium* eggs, Sarvel et al. [10] had earlier observed immature *S. mansoni* eggs that they considered viable, fluorescing when the same probe (Hoechst 33258) was used (a staining characteristic they considered to be observed only in dead eggs). 

The slight difference in the outcomes of the vital stains, where, for example, the 0.4% trypan blue staining looked reliable in the current study but had been previously been described as a poor tool for differentiating live and dead eggs [10], could be due to the difference in the types and source of samples, as well as the species of schistosoma used. However, it is beyond the scope of the current study to explain the probable mechanisms for the difference observed in the two species (*Schistosoma haematobium* and *S. mansoni*), as well as the type (urine and stool) and source (humans and mice) of samples.

Urogenital schistosomiasis is common in Ghana [20] and other sub-Saharan African countries [21]; thus, studies on *S. haematobium* are very important.

## 5. Conclusions

In this study, the fluorescence Hoechst 33258 provided a good staining outcome for differentiation between live and dead eggs, followed by 0.4% trypan blue staining where dead eggs retained the stains. Even though the 1% neutral red stain provided some evidence for which an egg could be viable or dead, the distinction was not very clear; therefore, it would be more useful when used in combination with other stains for egg viability determination. The benefits of the outcome of this study will include assessing the effect of drugs on *S. haematobium* eggs in schistosomiasis research.

The strength of this study is that clinical samples collected from school children were used. Additionally, the current study focused on *S. haematobium*, which is primarily responsible for urogenital schistosomiasis.

The limitation of this study is that the performance of the stains on the different stages of the *S. haematobium* in the human urine samples was not investigated.
